# Enhanced Infrared Sparse Pattern Extraction and Usage for Impact Evaluation of Basalt-Carbon Hybrid Composites by Pulsed Thermography

**DOI:** 10.3390/s20247159

**Published:** 2020-12-14

**Authors:** Jue Hu, Hai Zhang, Stefano Sfarra, Claudia Sergi, Stefano Perilli, Clemente Ibarra-Castanedo, Guiyun Tian, Xavier Maldague

**Affiliations:** 1 School of Automation Engineering, University of Electronic Science and Technology of China, Chengdu 611731, China; jue.hu.2@ulaval.ca (J.H.); g.y.tian@uestc.edu.cn (G.T.); 2 Department of Electrical and Computer Engineering, Computer Vision and Systems Laboratory, Laval University, QC G1V 0A6, Canada; clemente.ibarra-castanedo@gel.ulaval.ca (C.I.-C.); xavier.maldague@gel.ulaval.ca (X.M.); 3 Department of Industrial and Information Engineering and Economics (DIIIE), University of L’Aquila, Piazzale E. Pontieri 1, Monteluco di Roio, 67100 L’Aquila, Italy; stefano.sfarra@univaq.it (S.S.); stefano.perilli@univaq.it (S.P.); 4 Department of Chemical Engineering Materials Environment, Sapienza-Università di Roma & UdR INSTM, Via Eudossiana 18, 00184 Roma, Italy; claudia.sergi@uniroma1.it

**Keywords:** non-destructive testing, infrared thermography, hybrid composites, sparse pattern extraction

## Abstract

Nowadays, infrared thermography, as a widely used non-destructive testing method, is increasingly studied for impact evaluation of composite structures. Sparse pattern extraction is attracting increasing attention as an advanced post-processing method. In this paper, an enhanced sparse pattern extraction framework is presented for thermographic sequence processing and defect detection. This framework adapts cropping operator and typical component extraction as a preprocessing step to reduce the dimensions of raw data and applies sparse pattern extraction algorithms to enhance the contrast on the defect area. Different cases are studied involving several defects in four basalt-carbon hybrid fiber-reinforced polymer composite laminates. Finally, comparative analysis with intensity distribution is carried out to verify the effectiveness of contrast enhancement using this framework.

## 1. Introduction

Composite materials have been widely implemented in aircraft, aerospace, vehicles, and ships due to their prominent weight reduction and material enhancement properties. However, defects produced at the manufacturing stage and in-service will create high-security risks, especially when they appear on essential components. In order to address this issue, non-destructive testing techniques for inspection and evaluation have attracted increasing interest in recent years [1,2]. X-ray has a strong capability to provide promising results, but the potentially dangerous effects of ionizing radiation and the high price of the equipment restricts application. Conventional ultrasonic scanning is another established technique for composite inspection considering its reliability and low cost. However, its dependence on a coupling medium such as water causes limitations in the application of this inspection technique on waterproof materials, making it difficult for natural fiber composite inspection. In addition, these systems are typically not fast and are generally not appropriate for in-service inspections where access is limited to a single side [3].

Infrared thermography (IRT), as a widely used non-destructive testing method, has been increasingly studied as an alternative owing to its advantages, such as contactless inspecting mechanism, high spatial resolution, fast inspection rate, as well as advanced thermographic imaging techniques. IRT involves different thermal excitation methods, e.g., optical excitation thermography (OET), laser excitation thermography, vibrothermography (VT), and induction thermography [4]. In OET, the surface of the sample is heated by a flash lamp array and halogen lamps. Meanwhile, the thermal sequence of the subsequent cooling stage of the surface is recorded by a thermal camera. OET is a non-contact technique with a relatively large inspecting area [5,6]. Laser excitation thermography uses a laser beam as a thermal source. This excitation method is more suitable for micro-sized flaw detection tasks in composites, considering the structural complexity, which leads to abnormal thermal diffusion when conventional IRT techniques are adapted [7,8]. In VT, the sample is excited by an elastic wave from mechanical vibration. The mechanical energy is converted into thermal energy during the propagation of the damp acoustic waves, while energy dissipation in the vicinity of defects is more obvious due to the friction at the surrounding area [9,10,11]. Induction thermography is a non-contact, real-time measurement method over a large region, which is effective for the evaluation of composites with electrical conductivity, such as carbon fiber reinforced plastic (CFRP) [12,13]. Apart from studies on imaging techniques, extensive research on infrared processing methods have also been carried out. Advanced signal processing plays an essential role in IRT owing to its capability to extract latent information from high dimension data and enhance the contrast, which ultimately leads to an increased probability of detection and reduction in false alarm rate. The thermal signal reconstruction (TSR) algorithm is commonly implemented to reduce the noise level of thermal image sequences [14,15]. Principal component thermography has also been proposed as an infrared data processing technique to extract image features and reduce undesirable signals [16]. Pulsed phase thermography has been demonstrated as effective for spectral information mining in which amplitude and phase patterns could be extracted for further spectral analysis [17]. Nowadays, the virtual wave concept has become a popular topic in thermographic signal reconstruction [18,19]. This technique calculates the virtual wave from the measured temperature signal and then generates the output with an ultrasound reconstruction method, such as frequency domain synthetic aperture focusing technique (F-SAFT). Inspired by deep learning, the virtual wave reconstruction technique has been extended by adapting deep neural networks trained with synthetic data [20]. The performance of deep neural networks outperformed the model-based Stolt’s f-k migration method on both simulation and real-world data.

The sparse pattern extraction of data sequences has proven to be effective in latent representation learning, denoising, and data mining. Most natural signals exhibited sparsity properties in representations from adequate subspace. After the robust principal component analysis (PCA) problem was proposed for sparse pattern extraction for the first time [21], several attempts were made to solve this mathematical model effectively and efficiently [22]. These algorithms were adapted in a variety of applications, such as object detection [23], background separation [24,25], and image restoration [26]. In recent years, sparse pattern extraction has been studied as an advanced signal processing technique on IRT for non-destructive testing purposes. Promising performances have been achieved in both defect information enhancement and probability of detection improvement. Yan et al. [27] proposed a stable principal component pursuit (SPCP) algorithm for debonding detection in fiber-reinforced polymer composites. Experiments were carried out on two CFRP specimens with Teflon injection using pulsed thermography. The image quality of the results generated by this SPCP algorithm outperformed the conventional post-processing algorithms. Ahmed et al. [28] introduced an ensemble matrix factorization method for CFRP inspection. A sparse and deep layer decomposition was adapted for matrix factorization. The sparse decomposition and Gaussian filtering showed high performance on noise removal and defect information enhancement. The experiments were conducted on several CFPR samples using the OET system. The F-score evaluation of this algorithm revealed that it had promising defect detection capability and resistance to noise. In [29], Wu et al. proposed sparse PCT (SPCT) for surface defect detection of CFRP. An elastic net penalty was adopted in this SPCT algorithm, which improved the image quality around the defects compared with conventional PCT and SPCT with L_0_/L_1_ norm. Jie et al. [30] proposed a sparse moving window PCT (SMWPCT) algorithm to extend the SPCT algorithm with a moving window strategy. This algorithm revealed both correlations between the pixels in different regions and correlations in the same region at different sampling intervals. Results generated by SMWPCT on the CFRP board inspection were superior to several PCT variants.

In this work, an enhanced sparse pattern extraction framework is proposed for IRT sequence processing. This framework adopts the cropping operator and typical component extraction as a preprocessing step to reduce the dimension of raw data and applies sparse pattern extraction algorithms to enhance the contrast on the defect area. The experiments are carried out on four basalt-carbon hybrid fiber-reinforced polymer composite laminates, including sandwich-like (SAN) and intercalated (INT) structures with basalt-carbon-basalt (BCB) or carbon-basalt-carbon (CBC) stacking sequences, respectively. Different preprocessing methods and sparse pattern extraction algorithms are performed for comparative purposes in this framework. Specifically, the effectiveness of contrast enhancement using this framework is verified through the comparison of intensity distribution.

## 2. Specimens

**Figure 1:**
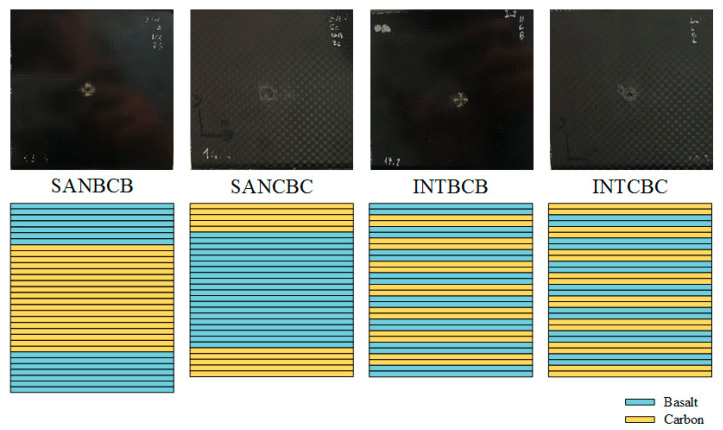
Photographs and schematic structures of the specimens.

All of the specimens involved in this study were manufactured with plain weave basalt (220 g/m^2^) and carbon (160 g/m^2^) fiber epoxy prepregs and formed in an autoclave using the same epoxy matrix (DT150). In the prepreg lay-up process, plain weave fiber layers were stacked with a 0/90° sequence. Then, during the forming process, all specimens were cured in SAN and INT structures, as shown in Table 1 and Figure 1.

During the high-velocity impact testing, the samples were mounted in a simply supported boundary condition along the edges using aluminum guides. The impact was performed using a helium gas gun with a spherical tempered steel projectile (mass = 1.725 g, diameter = 7.5 mm). In addition, the impact velocity (illustrated in Table 1) was obtained from a high-speed digital camera FASTCAM APX by Photron with a data acquisition system configured to 36,000 frames per second, as reported in [31].

**Figure 2:**
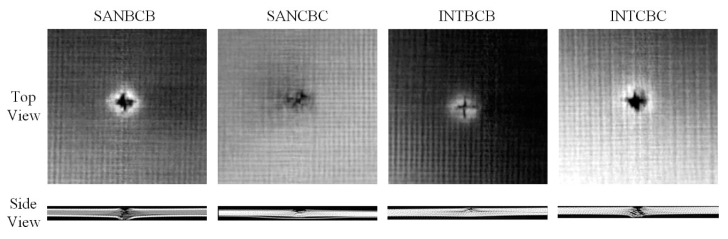
CT slices of the specimens.

Figure 2 illustrates the 100 μm resolution CT slices for the specimens. The CT slices were obtained using Siemens SOMATOM Definition AS+. The top view and side view slices were cropped near the impact location to provide a description of the damage induced by impact. It can be observed that delamination occurs on the sub-surface in the SAN structure, while more damage extends in the central thickness of the INT structure.

## 3. Methodology

### 3.1. Experimental Setup

As an optical excitation thermographic approach for IRT, pulsed thermography (PT) uses a flash lamps array with high-energy to generate uniform heating on the surface of specimens. After excitation, the heat transmits through the specimen by diffusion. As time elapses, the surface temperature decreases uniformly for a specimen without internal flaws. Conversely, surface and sub-surface discontinuities act as resistances to heat flow, which change the diffusion rate and produce abnormal temperature patterns. As a time-domain sequenced imaging approach, PT allows for the implementation of advanced imaging processing techniques to extract more visible imprints of defects.

**Figure 3:**
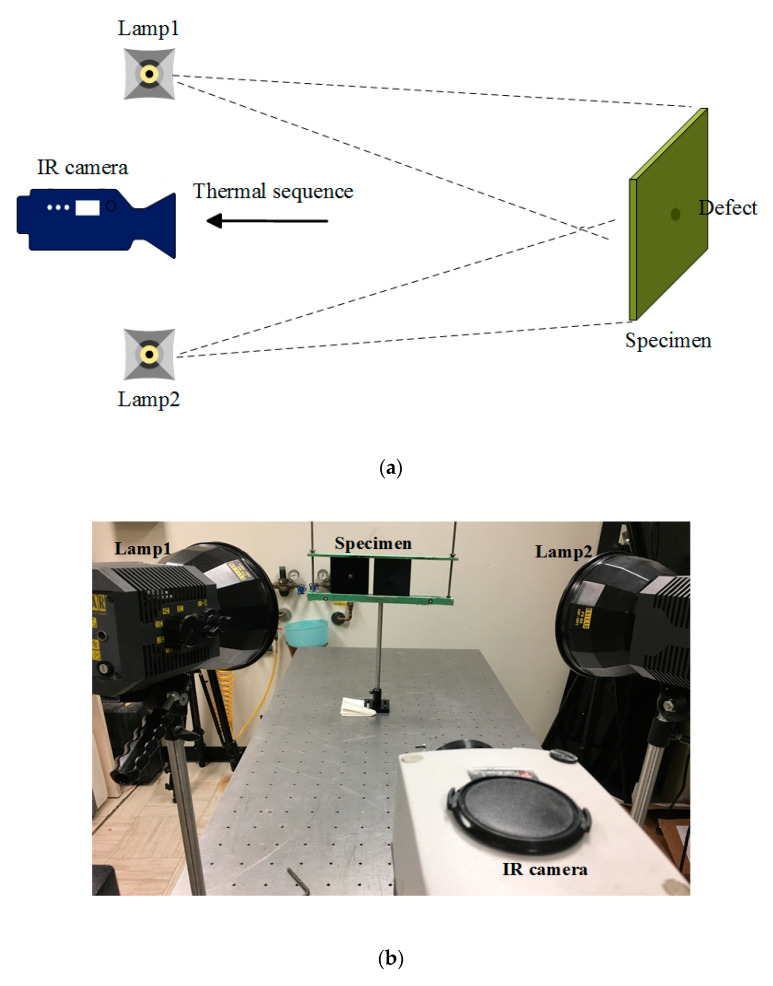
Optical excitation thermography inspection: (**a**) schematic configuration; (**b**) experimental setup.

Figure 3 shows the schematic configuration and experimental setup for PT. Two flash units (Balcar FX60, 6.4 kJ, 2 ms duration) were used to generate optical flashes in this configuration. A mid-wave IR camera (FLIR Phoenix) with a frame rate of ~55 Hz and an NETD of 25 mK was adapted to record the temperature profile. The cooling time was set at 10 s for flashes. The camera spatial resolution was 640 × 512 pixels (25 μm × 25 μm of detector size), and a 50 mm lens was employed to provide a field of view (FOV) of 18.2° (horizontal) × 14.6° (vertical). Hence, from a distance of 50 cm between the camera and specimens, the FOV was 16.0 cm (horizontal) × 12.8 cm (vertical), which corresponded to 40 pixels/cm.

### 3.2. Sparse Pattern Extraction

**Figure 4:**
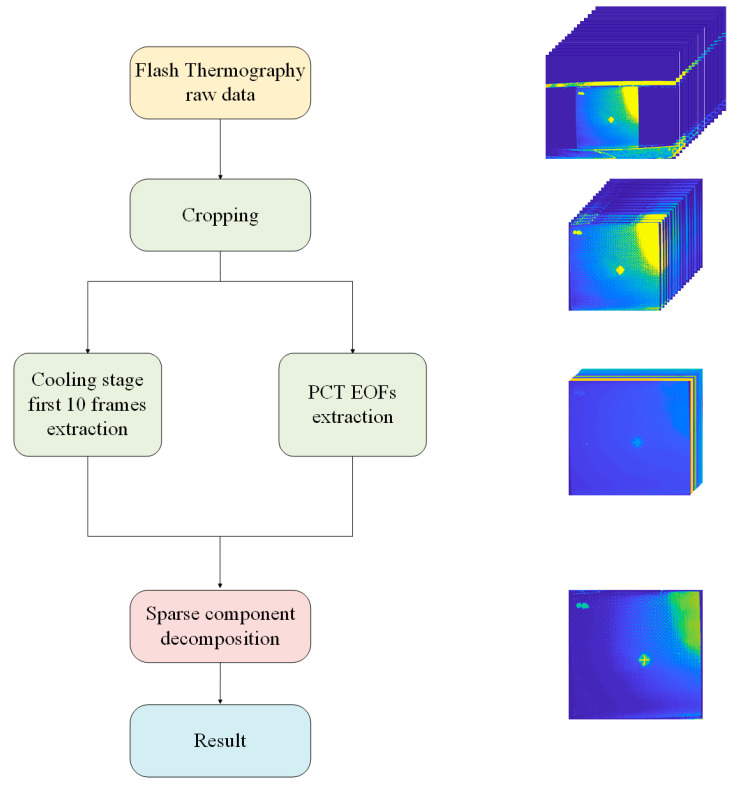
Enhanced infrared sparse pattern extraction framework for thermal sequence processing.

In this work, the whole signal processing framework based on sparse pattern extraction is illustrated in Figure 4. First, the thermal sequence was preprocessed to extract typical information. The preprocessing pipeline included a cropping operation and typical components extraction. Direct frame extraction was performed when the task required more on-surface information, while PCT was adopted when the task focused on defects appearing on sub-surface layers. PCT relies on singular value decomposition (SVD) to project data onto empirical orthogonal functions (EOFs). In addition, it is worth noting that the typical component extraction aims at dimension reduction in order to reduce the computational complexity of the sparse component extraction process. Then, sparse patterns are extracted with sparse component decomposition techniques based on different assumptions and constraints. In this work, sparse component decomposition techniques were adopted to separate the defect information from the background and thermal noises. The tensor was decomposed into low-rank and sparse components; the sparse component referred to the defect information, while the low-rank component was inherent to the background information. In the following parts of this section, we introduce several effective sparse component decomposition techniques that are involved in the experiments.

In sparse pattern extraction, the input data matrix $D\in R^{p\times f}$ is decomposed into several components using optimization methods as follows:

(1) $$\underset{A,E}{min}{\left\|A\right\|}_{*}+\lambda {\left\|E\right\|}_{1}\;\mathrm{subject}\;\mathrm{to}\;D=A+E+N$$

where ${\left\|\cdot \right\|}_{*}$ refers to the nuclear norm of a matrix, ${\left\|\cdot \right\|}_{1}$ denotes the sum of absolute values of matrix, A denotes the low-rank component, E denotes the sparse component, N represents the noise, λ is the positive weight parameter, p=m×n refers to the pixel number of each frame (m and n denote the vertical and horizontal dimension of each frame), and f refers to the frame number of the IRT data sequence.

Here, we introduce five state-of-the-art techniques for sparse pattern extraction, which are incorporated into the proposed sparse pattern extraction framework for experiments on composite laminates.

The augmented Lagrange multipliers (ALM) algorithm tackles the sparse pattern extraction problem through minimizing the Lagrange function:

(2) $$L\left(A,E,Y,\mu \right)={\left\|A\right\|}_{*}+\lambda {\left\|E\right\|}_{1}+\langle Y,D-A-E\rangle +\frac{\mu }{2}{\left\|D-A-E\right\|}_{F}^{2}$$

which imports the Lagrange multiplier Y and penalty parameter μ [22]. This algorithm achieves a superior convergence property and, at the same time, has the advantages of simple parameter adjustment. The exact ALM (EALM) algorithm minimizes the Lagrange function for each iteration, while the inexact ALM (IALM), which is also known as the alternating direction method (ADM), updates components A and E until convergence. Compared with EALM, IALM further reduces the computational complexity.

The variational Bayesian tensor factorization (VBTF) algorithm uses variational Bayes as an approximation method for the inference on the joint distribution p(D,P,Q,E,γ,α,β) to complete the sparse pattern extraction task [32]. The joint distribution is expressed as:

(3) p(D,P,Q,E,γ,α,β)=p(D|P,Q,E,β)p(P|γ)p(Q|γ)×p(E|α)p(γ)p(α)p(β)

The low-rank component A is expressed by the product of two-factor matrixes as $A=PQ^{T}$. The γ denotes inverse variance with conjugate Gamma hyperpriors. The precision α and noise precision β are both set with Jeffreys prior.

Probabilistic robust matrix factorization (PRMF) is proposed to form the sparse pattern extraction task as a maximum a posteriori (MAP) estimation problem [33]. The sparse component E is decomposed as $E=PQ^{T}$. The data matrix D is factorized as $D=PQ^{T}+A$ and some probabilistic assumptions are made as follows:

(4) $$p_{ij}|\lambda_{p}\sim N(p_{ij}|0,\lambda_{p}^{-1})$$

(5) $$q_{ij}|\lambda_{q}\sim N(q_{ij}|0,\lambda_{q}^{-1})$$

The joint distribution is:

(6) $$p(P,Q|D,\lambda_{p},\lambda_{q},\lambda )\propto p\left(D|P,Q,\lambda \right)p\left(P|\lambda_{p}\right)p(Q|\lambda_{q})$$

where $\lambda_{p}$,$\lambda_{q}$, and λ are hyperparameters. Then, the log-likelihood formulation is shown as:

(7) $$logp\left(P,Q|D,\lambda_{p},\lambda_{q},\lambda \right)=-\lambda {\left\|D-PQ^{T}\right\|}_{1}-\frac{\lambda_{p}}{2}{\left\|P\right\|}_{2}^{2}-\frac{\lambda_{q}}{2}{\left\|Q\right\|}_{2}^{2}+C$$

where C is a constant. Maximizing the joint distribution is the same as minimizing Equation (8):

(8) $$\underset{P,Q}{min}{\left\|D-PQ^{T}\right\|}_{1}+\lambda_{p}{\left\|P\right\|}_{2}^{2}+\lambda_{q}{\left\|Q\right\|}_{2}^{2}$$

The conditional EM (CEM) algorithm is used to update two factor matrixes P and Q individually.

Similar to VBTF and PRMF, the mixture of Gaussian (MoG) completes the sparse pattern extraction task by building a probabilistic model [25]. The low-rank component A is decomposed as $A=PQ^{T}$. CEM is also adapted in the MoG model for parameter updating. The objective function L(π,σ,P,Q) is shown as:

(9) $$L\left(\pi ,\sigma ,P,Q\right)=-ln\;p(D|\pi ,\sigma ,P,Q)+R_{F}\left(\pi ,\sigma \right)+R_{B}\left(P\right)$$

which is constructed with the likelihood term and the regularized terms, where π denotes the mixture rate and σ denotes variance in the MoG model. The regularized term $R_{F}\left(\pi ,\sigma \right)$ refers to noise distribution. The regularized term $R_{B}\left(P\right)$ corresponds to the Mahalanobis distance between each row vector of P to that of P in the previous iteration. Thus, this regularized term rectifies the current learned subspace by the previously learned one.

Improved robust tensor principal component analysis (IRTPCA) is proposed for three-dimension tensor factorization [34]. The input data in this model is different from all of the previous algorithms and is formatted as tensor $D\in R^{m\times n\times f}.$ IRTPCA is designed on the base of tensor (t)-SVD and adopts ADM for optimization. t-SVD is performed on the tensor to calculate the singular value tensor. Then, the singular value tensor is flattened and processed with singular value thresholding (SVT). The outputs of SVT are reconstructed into tensors and used to update the low-rank component A and sparse component E.

### 3.3. Ensemble Sparse Pattern Extraction

Multilayer ensemble architecture has recently been proved to be useful for improving the performance of low-rank and sparse component factorization. The ensemble structure can be denoted as follows:

(10) $$D=A_{1}+E_{1}+N_{1}\;A_{1}=A_{2}+E_{2}+N_{2}\;A_{2}=A_{3}+E_{3}+N_{3}\;\cdots \;A_{k-1}=A_{k}+E_{k}+N_{k}$$

In the first layer, the data can be decomposed into a low-rank component, a sparse component, and noise by using the sparse pattern extraction algorithm introduced previously. Repeating this procedure will progressively extract the information. In the next layer, the parameter updating follows the same rule defined in the sparse pattern extraction algorithm while the input is substituted by the low-rank component of the previous layer. Then, the final representation can be expressed as:

(11) $$D=A_{k}+\overset{k}{\underset{i=1}{\sum }}E_{i}+\overset{k}{\underset{i=1}{\sum }}N_{i}$$

where $A_{k}$ refers to the low-rank component of the final layer, $E_{i}$ and $N_{i}$ denote the i-th sparse component and noise, and k denotes the number of layers.

## 4. Results and Discussion

**Figure 5:**
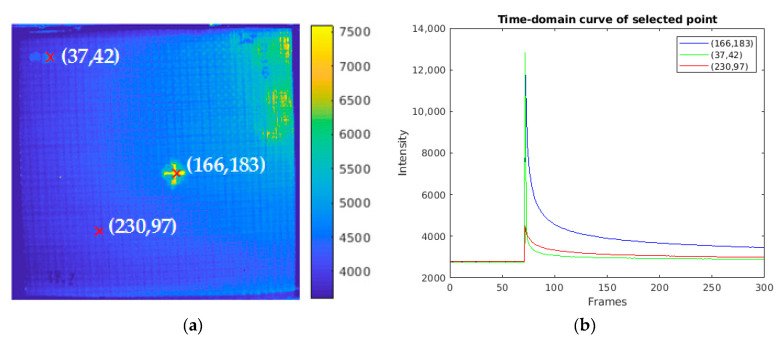
Thermographic result and analysis for the intercalated structure with basalt-carbon-basalt (INTBCB) specimen: (**a**) pseudo color image of the typical frame; (**b**) temperature profile curve of the selected points.

**Figure 6:**
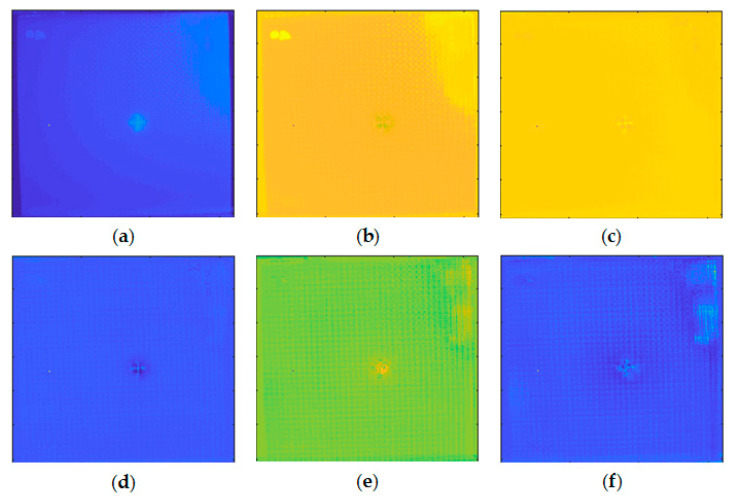
PCT results of the thermal sequence conducted through flash thermography on the INTBCB specimen: (**a**) EOF 1; (**b**) EOF 2; (**c**) EOF 3; (**d**) EOF 4; (**e**) EOF 5; (**f**) EOF 6.

Figure 5a shows the pseudo color image of the frame extracted from the thermal sequence recording the flash thermography experiment on the INTBCB specimen. The extracted frame is the frame following the peak temperature (flash excitation), which has a high contrast of the impact-induced surface damage area and the normal area without impact. It is obvious that the cross-shape surface crack induced by impact could be found on this frame due to the different thermal conductivity on the composite material and air remaining in the crack. Figure 5b illustrates the temporal temperature profile on points from different locations. The difference existing in the temporal temperature profile is the base for further signal processing algorithm implementation, which aims at enhancing the spatial contrast. Figure 6 presents the PCT results of the thermal sequence. Six EOFs extracted by PCT from the thermal sequence conducted through flash thermography are shown. It is clear that the surface crack information is recorded in EOF 3 and 4, while the EOF 2 and 6 contain the orientation information of the woven fiber. EOF 5 records the impact-induced damage caused in greater depth. EOF 1, as the main component, merges all of the information described above. In the PCT results, some structural patterns and defect information could be preliminarily observed. However, these patterns are scattered in several components. This indicates that PCT is suitable as a data preprocessing step. Sparse component decomposition is then adopted to integrate the information with sparse features to obtain high contrast outputs.

**Figure 7:**
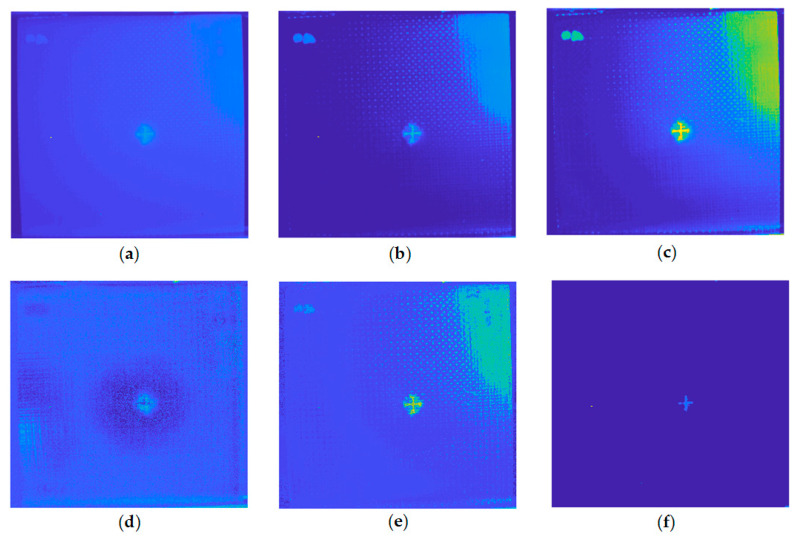
Comparison on the enhanced sparse pattern extraction results of the INTBCB specimen: (**a**) PCT; (**b**) IALM; (**c**) VBTF; (**d**) PRMF; (**e**) MoG; (**f**) IRTPCA.

Figure 7 shows the enhanced sparse pattern extraction results of the INTBCB specimen. It can be observed that IALM, VBTF, and MoG achieve high contrast between the impact-induced damage and the surrounding area. Only the PCT processing result (Figure 7a) shows the impact-induced surface crack and damage while the contrast on the surface information is low. This is due to the lack of further sparse component extraction processing. Similar to PCT, single layer PRMF (Figure 7d) extracts less sparse information than the other two probabilistic models because the different prior distribution used in the model influences the effectiveness of specific implementation. However, the effectiveness could be boosted through the multilayer ensemble structure, which will be discussed in the following experiments. IRTPCA (Figure 7f) only extracts the pure surface crack information, while other surface information, such as orientation information of woven fiber, will be removed. This is mainly due to the adoption of the thresholding method in the approximation process.

**Figure 8:**
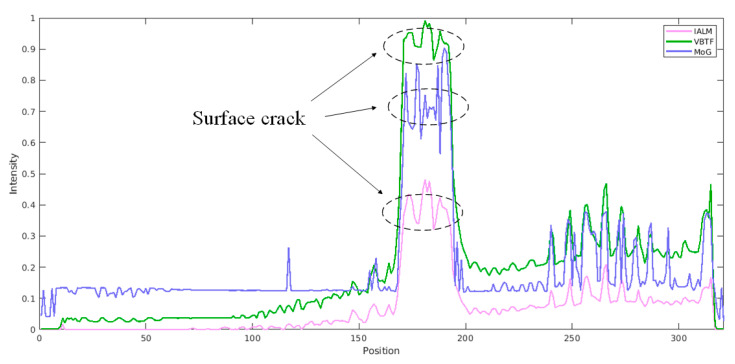
Intensity distribution curve on the INTBCB specimen acquired by different processing methods.

**Figure 9:**
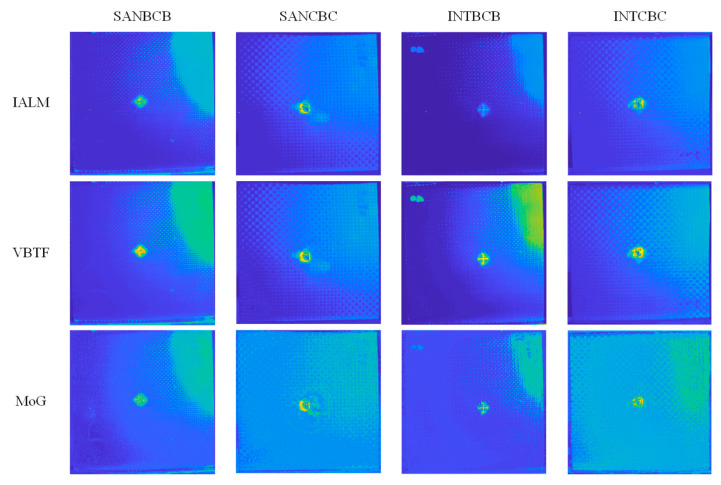
Comparison on three high-performance algorithms.

In order to provide a more precise comparison, three sparse pattern extraction methods with significantly superior performance than those shown in Figure 7 were chosen for further intensity distribution and contrast analysis. Figure 8 illustrates the intensity distribution acquired by different processing methods with a fixed horizontal location *x* = 167 and variant vertical position. It can be concluded from this intensity distribution curve that the VBTF processing result shows the highest contrast on the surface crack area compared with other sparse pattern extraction methods. In addition, comparison results on all four kinds of hybrid fiber-reinforced laminates are illustrated in Figure 9. It is worth noting that all three effective algorithms involved in this comparison extract the surface crack, impact-induced damage, and surface fiber orientation successfully. Moreover, the enhanced VBTF model shows even higher contrast on the surface information and is proven to be more suitable for the implementation on the enhanced infrared sparse pattern extraction framework in general laminates evaluation. On SANCBC and INTCBC samples, the enhanced MoG algorithm improves the contrast on the defect area, while the fiber orientation and composite structural information are not preserved. The defect information extraction and background interference elimination capabilities make the enhanced MoG algorithm more suitable for the detection tasks of sub-surface defects in complex surface situation and great depth. All in all, these three enhanced sparse component decomposition algorithms have their own applicable scenarios in composite inspection using IRT. The practical industrial implementation requirements according to the specific detection task must be taken into account when selecting the appropriate algorithm.

**Figure 10:**
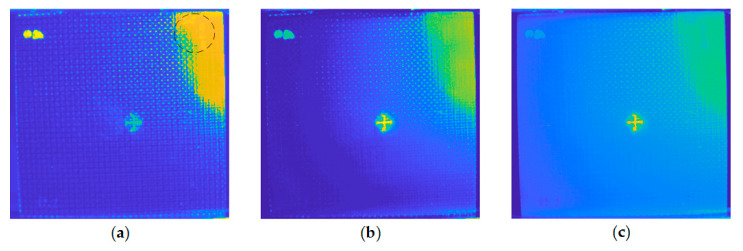
Comparison of the different preprocessing implementation details using the INTBCB specimen: (**a**) Cropping and first 10 frames extraction in the cooling stage; (**b**) Cropping and 10 principal components extraction; (**c**) Without cropping and 10 principal components extraction.

**Figure 11:**
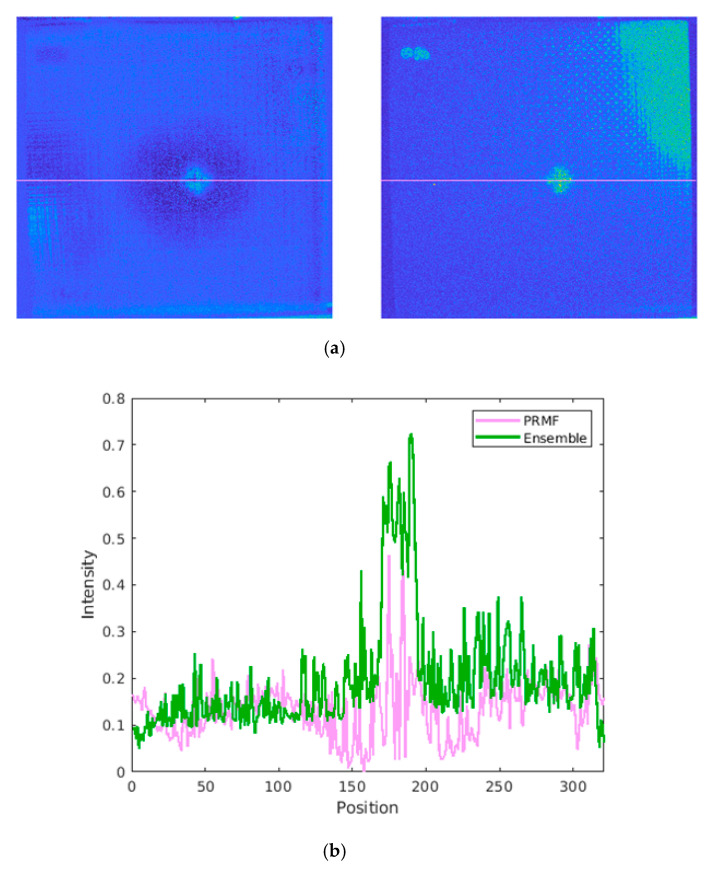
Comparison of the ensemble structure implementation using the INTBCB specimen: (**a**) Performance comparison on PRMF and ensemble PRMF; (**b**) Intensity distribution curve.

For further implementation details, a comparison was conducted to exhibit the significance of the operations adapted in the preprocessing pipeline. The results shown in Figure 10 were all processed with the enhanced VBTF model, while different preprocessing techniques were conducted. As shown in Figure 10a,b, direct frame extraction performed in the cooling stage extracts more surface information, e.g., the name of the specimen marked by ink on the upper right corner and high contrast fiber orientation. PCT extracts both information on the surface and sub-surface, while the contrast of surface information is lower than direct frame extraction. It could be concluded that the choice of a typical component extraction method depends on the actual industrial application requirements. As shown in Figure 10b and c, the result provided by the enhanced sparse pattern extraction algorithm with the cropping operation shows higher contrast on the surface orientation of woven fiber. The cropping operation deletes the background information, which is in the FOV but outside of the specimen boundary. This prevents the sparse noise in this background information from influencing the contrast of the output image quality. In Figure 11, the multilayer ensemble structure is performed with the PRMF algorithm on the INTBCB specimen. Figure 11a shows that the ensemble PRMF extracts more fiber orientation information into the sparse component and improves the contrast on the surface crack induced by impact. Single-layer PRMF only extracts a small portion of the defect information, while the ensemble PRMF conducts deep layer decomposition on the data after preprocessing. Through the multilayer structure, the sparse patterns in the data are repeatedly mined. This ultimately results in a significant improvement in the contrast of the defect area. The intensity distribution curve illustrated in Figure 11b further proves the effectiveness of the multilayer ensemble architecture. The multilayer ensemble PRMF improves the contrast on the impact-induced damage area compared to single-layer PRMF. Observing the intensity distribution curve, it is obvious that the variation of the curve between the positions 150 and 200 refers to the signal contrast of the defect area. The curve referring to the result of the ensemble PRMF (green line in Figure 11b) shows a more drastic variation near the defect area than the curve indicating the result of single-layer PRMF (pink line in Figure 11b). It could be summarized that the ensemble architecture has the capability of improving the sparse pattern extraction performance prominently while slightly increasing computational cost. This provides new inspiration for designing IRT signal processing algorithms, i.e., sacrificing the computational efficiency to enhance the defect information. The balance of output image quality and computational complexity should be considered according to the actual industrial implementation.

**Figure 12:**
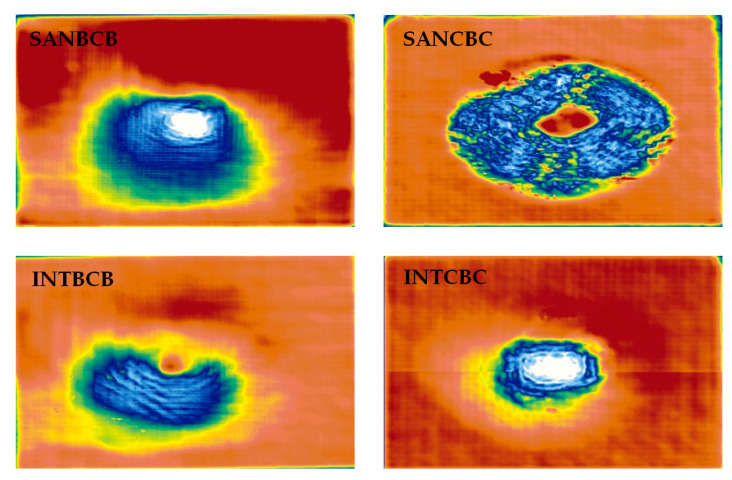
Validation using ultrasonic testing.

In order to verify the efficacy of this enhanced infrared sparse pattern extraction framework, some widely used non-destructive testing methods were involved for comparative purposes. Ultrasonic testing was performed (illustrated in Figure 12). A probe (Olympus Panametrics-NDT A310S, 5 MHz) was adapted to induce the elastic wave and receive the echo. An Olympus OmniScan MX was used for data processing and visualization. A robotic GLIDER X-Y Scanner was used for raster scanning, and a water tank was used to contain the coupling medium required for this experiment. In Figure 12, the results of four composite laminates are shown. Compared with the proposed enhanced infrared sparse pattern extraction framework, the results of ultrasonic testing have the characteristic of low spatial resolution. From the results, the existence of impact-induced damage can be detected, while the morphology of impact-induced damage cannot be specifically observed due to the limitation of low spatial resolution. Interestingly, in the SANCBC sample, the delamination around the impact-induced damage is completely revealed by ultrasonic testing. This delamination is identified in the CT results (side view of SANCBC in Figure 2), which are near the other side of the impact surface. Moreover, in the other three samples, the heterogeneous distribution of resin can be observed in the results of the ultrasonic testing. The delamination information is also revealed in the results of the proposed framework (in Figure 9), while the completeness is not better than the results produced by ultrasonic testing. Unfortunately, the resin-rich area is not detected in the results of the proposed framework due to the limitation of thermal energy excited by the flash lamp. Therefore, in practical industrial implementation, the proposed framework is more suitable for high-speed preliminary detection of surface or subsurface defects. In subsequent work, the defective samples confirmed by high-speed preliminary detection will be subjected to ultrasonic testing for further delamination detection and internal structure analysis.

**Figure 13:**
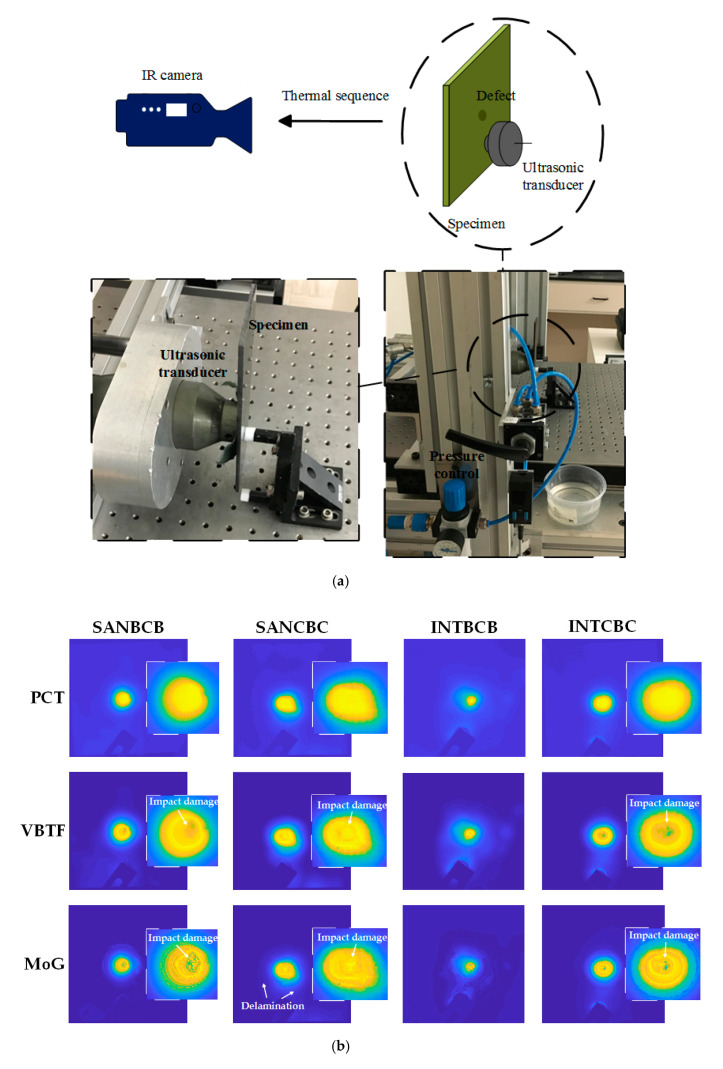

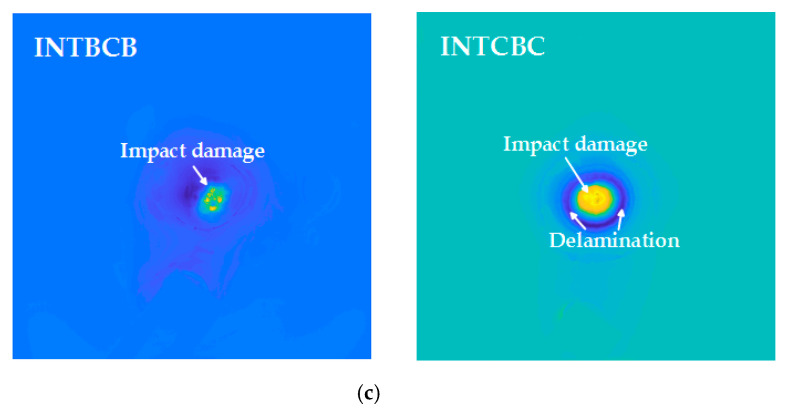
Validation using vibrothermography on four composite laminates: (**a**) Schematic configuration of vibrothermography; (**b**) Data processing results of PCT, enhanced sparse pattern extraction using VBTF and enhanced sparse pattern extraction using MoG; (**c**) The second sparse pattern extracted with MoG from the INTBCB and INTCBC specimens.

Vibrothermography was also conducted (shown in Figure 13). The same IR camera as PT was used in the vibrothermography experiment with the same frame rate (55 Hz). The transducer was pressed against the specimen, and two periods of 0.2 Hz (10 s) lock-in ultrasonic waves were delivered. In Figure 13b, data processing results of four composite laminates are shown. Owing to the excitation method, vibrothermography has a stronger capability on internal structure inspection than PT. Correspondingly, this kind of IRT approach sacrifices the surface and sub-surface information compared with PT. The PCT results of all of the specimens only revealed the location of the impacted area, while neither the morphology of the impact-induced damage nor the fiber orientation was inspected. After processing with the proposed enhanced sparse pattern extraction framework using VBTF and MoG, part of the surface and sub-surface defect information was extracted into the sparse pattern, e.g., impact-induced damage in rhombus shape in the SANBCB specimen, damage in the shape of a circle in the SANCBC specimen, and circular damage in the INTCBC specimen. This is particularly evident in the zoom-in enlarged figure of the impacted area in Figure 13. It is worth noting that the proposed enhanced infrared sparse pattern extraction framework has robustness and generalization capabilities, which could also improve the defect information from another modality of thermal excitation. As discussed before, the enhanced MoG algorithm is more suitable for sub-surface defect detection. It could be combined with the characteristics of vibrothermography to further extract the internal structure information. Thus, the enhanced MoG algorithm is a preferred choice for vibrothermography data processing. Two interesting points can be further observed in the results of enhanced infrared sparse pattern extraction using MoG. One is the delamination around the impact-induced damage area in the SANCBC specimen (clearly indicated in the ultrasonic testing results, Figure 12) that is revealed in the processing result of enhanced MoG, as shown in Figure 13b. Another aspect is the fact that the second sparse pattern extracted with the enhanced MoG contains higher contrast impact-induced damage morphology information. In the previous experiments, especially those using PT as the thermal excitation modality, the main sparse patterns were selected as the results of the enhanced sparse pattern extraction framework because the impact-induced damage always existed in the pattern with the highest sparsity. However, the second sparse pattern extracted with the enhanced MoG successfully revealed the defect information, which was not presented in the main sparse pattern of INTBCB and INTCBC (shown in Figure 13c). The reasonable explanation of this phenomenon is that vibrothermography mainly extracts internal and sub-surface information, while extremely limited surface information can be gathered in original data. This results in the main sparse pattern concentrating on the internal structure and limited surface information being distributed into the second sparse pattern. To summarize, the enhanced MoG on vibrothermography data could reveal sub-surface and internal information of composite laminates. This also provides effective information compensation for PT in practical industrial inspection tasks.

## 5. Conclusions

In this paper, an enhanced sparse pattern extraction framework was presented for IRT sequence processing. Different cases were studied involving several defects in four basalt-carbon hybrid fiber-reinforced polymer composite laminates. In the preprocessing step of this enhanced sparse pattern extraction framework, cropping reduced the background sparse noise influence on the result quality. Using frame extraction in the cooling stage, or EOFs in PCT, depends on the actual industrial application requirements of surface or sub-surface evaluation. The experiments and comparative analysis verified the contrast enhancement ability of this framework on hybrid fiber-reinforced laminate inspection. Moreover, the comparative study proved that IALM, VBTF, and MoG were more suitable for defect contrast enhancement on the IRT sequences collected from these composite specimens. It is worth mentioning that the VBTF algorithm showed the most promising performance of defect information extraction according to the intensity distribution. Another interesting finding was that the ensemble framework could effectively improve the performance of some sparse pattern extraction algorithms, which could provide new inspiration for designing IRT signal processing algorithms. In order to further verify the efficacy of the proposed enhanced infrared sparse pattern extraction framework, ultrasonic testing and vibrothermography were employed for comparative purposes. Data processing results on vibrothermography showed that the enhanced MoG revealed sub-surface and internal structure information. This proved that the proposed framework had robustness and generalization capabilities.

## Figures and Tables

**Table 1:** Summary of the specimens.

Structure	Stacking Sequence	Fiber Volume Fraction	$\mathrm{Density}\;[g/cm^{3}]$	Thickness [mm]	Impact Velocity [m/s]
SANBCB	${\left[B_{7}/C_{9}\right]}_{S}$	0.61 ± 0.01	1.72 ± 0.02	4.40 ± 0.06	201.69
SANCBC	${\left[C_{5}/B_{10}\right]}_{S}$	0.63 ± 0.01	1.87 ± 0.02	4.00 ± 0.05	251.71
INTBCB	${\left[{\left(B_{2}/C_{2}\right)}_{3}/B_{2}/C\right]}_{S}$	0.61 ± 0.01	1.77 ± 0.02	4.20 ± 0.05	248.22
INTCBC	${\left[{\left(C_{2}/B_{2}\right)}_{3}/C_{2}/B\right]}_{S}$	0.60 ± 0.01	1.71 ± 0.01	4.30 ± 0.05	253.46
